# Development of an intelligent system based on metaverse learning for students with disabilities 

**DOI:** 10.3389/frobt.2022.1006921

**Published:** 2022-12-06

**Authors:** Souhir Sghaier, Abir Osman Elfakki, Abdullah Alhumaidi Alotaibi

**Affiliations:** Department of Science and Technology, College of Ranyah, Taif University, Taif, Saudi Arabia

**Keywords:** e-learning, 3D virtual world, metaverse, intelligent system, artificial intelligence

## Abstract

Due to the coronavirus-2019 pandemic, people have had to work and study using the Internet such that the strengthened metaverse has become a part of the lives of people worldwide. The advent of technology linking the real and virtual worlds has facilitated the transmission of spatial audio and haptics to allow the metaverse to offer multisensory experiences in diverse fields, especially in teaching. The main idea of the proposed project is the development of a simple intelligent system for meta-learning. The suggested system should be self-configurable according to the different users of the metaverse. We aimed to design and create a virtual learning environment using Open Simulator based on a 3D virtual environment and simulation of the real-world environment. We then connected this environment to a learning management system (Moodle) through technology for 3D virtual environments (Sloodle) to allow the management of students, especially those with different abilities, and followed up on their activities, tests, and exams. This environment also has the advantage of storing educational content. We evaluated the performance of the Open Simulator in both standalone and grid modes based on the login times. The result showed times the standalone and grid modes of 12 s and 16 s, which demonstrated the robustness of the proposed platform. We also tested the system on 50 disabled learners, according to the *t*-test of independent samples. A test was conducted in the mathematics course, in which the students were divided into two equal groups (n = 25 each) to take the test traditionally and using the chair test tool, which is one of the most important tools of the Sloodle technology. According to the results, the null hypothesis was rejected, and we accepted the alternative hypothesis that demonstrated a difference in achievement between the two groups.

## Introduction

Virtual worlds or environments are defined as programs that represent, build, and simulate three-dimensional virtual environments. Users can create virtual characters called ‘avatars’ that embody them. The experience can be applied to children and adults (Cadet et al., 2022). Users can also build and design buildings and models and perform various types of activities. Virtual environments are characterized by realism and provide interaction and integration or indulgence (immersive) for users. In addition, relationships and links between students and teachers worldwide can be created in any course, even theoretically or practically, in virtual laboratories, like computer science, medicine, physics, chemistry (Reeves et al., 2021), and earth science (Chang et al., 2020). However, teachers must understand these virtual (three-dimensional) environments and discover the best methods of improving performance exercises and educational activities. These educational activities can be designed and delivered within the content of these three-dimensional environments. However, virtual worlds cannot manage learners’ systems, manage students’ records, and track activities. Moreover, these worlds cannot store some types of educational resources because they are not designed for this purpose. Therefore, the next trend in e-learning is to integrate virtual worlds with learning management systems. Such collaborative and cooperative teaching opportunities can improve the performance of education. However, the learning and spatial abilities of learners in real and virtual environments differ significantly (Sun et al., 2019).

“Virtual teacher” is expected to become an actual job description, with the availability of innovations such as virtual headphones, smart glasses, applications for remote classroom management, and other technologies that will shape education.

The advantages of learning in a 3D virtual learning environment (VLE) include assistance and support in building knowledge, integration into the virtual environment, interactive learning, and training. These rich multimedia spaces fundamentally change the way we think about learning, social interaction, and self-expression. VLEs encourage and stimulate their use in web platforms because of their technical flattening (controlling the shape of the surface and the environment). Therefore, the combination of these two learning methods; i.e., VLE and learning systems (LSs), allows developers and educators to discover new ways of learning across web platforms and in multi-user virtual (MUV) environments.

This new technology may impose on us a new life with a virtual world that is inseparable from the reality that we live in. The spatial orientation skills in real and virtual locations differ between these two environments, as demonstrated by Pastel et al. (2022) and Kober et al. (2021).

Recently, people worldwide can communicate through augmented virtual worlds in many fields, for instance in the social learning space, as described by Scavarelli et al. (2021), or using electrical equipment in an augmented reality world as reported by Westerfield et al. (2015), hence further reducing distances. Furthermore, the word “metaverse” is becoming a ubiquitous term to describe how people may walk and talk in their daily affairs. Indeed, the metaverse is related to the creation of an integrated virtual world that aims to develop virtual reality technologies to unprecedented levels. These new terms refer to the products and releases that are expected to appear soon. Many fantasies appearing in science fiction films (Old Testament metaverses) will soon become reality. We could live in the future as if we were already in it. The metaverse is based on three basic and main aspects that distinguish it from the Internet:

### Existence

Individuals are present in virtual spaces and interact with others as if they were with them in the same place. The concept of being present relies on embodiment, which improves the quality of interaction over that provided by the Internet.

### Interoperability

Interoperability is a feature of the metaverse that allows individuals to easily, smoothly, and instantaneously move to any place and space in the virtual world, using available avatars and digital elements.

### Standardization

Standardization allows users to interoperate services and systems in an integrated manner through the metaverse, including media technologies, through the immersive virtual environment, as described by Meyer et al. (2019), by sending text messages to the printing press, where the usual technological standards are adopted collectively for all means. This applies to international organizations such as OMIG.

The metaverse allows students with disabilities to experience a situation or a scene while they are in their classroom through 3D scenes, with sound effects synchronized with the image. When the students move their heads, they will notice that the world is protectively revolving around them, giving them a feeling that this scene is real.

This study describes the design and construction of an environment integrated with VLE management to manage the records and follow-up activities of students with disabilities that takes advantage of the ability of learning management systems to store educational content. The education of students with disabilities in the virtual world offers a hybrid method combining the advantages of open-source systems like Moodle and simulation software (Open Simulator), through Sloodle. These techniques help researchers to build and design VLEs and improve the quality of educational content in the learning management system. Moreover, these methods improve the interactions to support MUVs.

We propose a novel approach that aims to teach students with diverse disabilities (hyperactivity, impaired hearing, visual impairment, Impaired mobility, etc.) in a 3D virtual reality world that provides total learner immersion. This intelligent system aims to help students to improve their performance in any institution, even those with students without these disabilities. The proposed application was very acceptable by every student in general and especially by disabled students. Indeed, it minimized the cost of study and teaching, encouraged learners, and provided opportunities to learn and study in any location. Moreover, it encouraged learners to transcend passive reception of information and moves toward active participation in the education process. The proposed intelligent system allows all users to communicate with other residents of the environment by voice chat and SMS messages; residents can also receive calls from outside the virtual community. Moreover, they can travel in its various parts, socialize with other residents, participate in individual and group activities, and create virtual properties and services. The presented work not only helps in most parts of the integration of people with disabilities into normal life but also improves their level of education and their results. All these advantages specified our system, especially for disabled people.

## Related work

The evolution of technology, especially for e-learning (Mejbri et al., 2022), academic library applications (Rafique et al., 2021), automatic programming, and the application of artificial intelligence (Souai et al., 2022) have made the virtual world a reality^1^. Fully virtual study is another focus of future education, particularly for people with disabilities. Laabidi et al. (2014) reported the application of a learning application to people with disabilities based on Moodle^Acc+^. Another example is the Astra Nova virtual online school created by the entrepreneur Elon Musk, which accepts children from anywhere in the world from 8 to 14 years of age. At Astra Nova, students are encouraged to acquire and trade the “Astra” cryptocurrency within a system to reward students for good behavior and are taught about money management and entrepreneurship. Nowadays, we speak about the new term “metaverse”, which will be linked to an advanced stage that transcends the Internet in stages to provide virtual spaces where humans will integrate and experience augmented virtual reality. We apply this new technology in learning, which attracts particularly students with disabilities students with the idea of living and experiencing life in another world in a 3D space while they are in their place. This new technology provides a series of virtual worlds, featuring endless interactions between users through each user’s avatar. Thus, it is important to learn muscle movements, voice tones, emotions, and facial expressions of real announcers. Artificial intelligence examines the operator’s interactive patterns such as face recognition, as presented by Sghaier et al. (2018) and Ben Fredj et al. (2021), in addition to handwriting in the metaverse to express the user’s behavior and character. The metaverse applies artificial intelligence to the corresponding Avatar. Jeong et al. proposed to replace micro-teaching with metaverse learning using the Virbela platform. In this application, the metaverse used artificial intelligence to build human-like voices and unique content (Jeong et al., 2021). The metaverse aims to take virtual reality technologies to unprecedented levels and has recently announced its entry into this new world (second life) with a 3D environment. Consequently, the role of the user is not limited to looking at it on their screen, but rather entering and immersing themselves in this environment and becoming one of its elements. Indeed, the user’s senses are separated from their real worlds during their stays in the virtual world. Virtual environments have become of great importance in the field of education, which permits the building and creation of relationships and links between students and teachers. For example, UC Berkeley students created a campus inside the Minecraft game based on the metaverse and launched a virtual graduation ceremony by applying artificial intelligence in the context of the coronavirus disease 2019 pandemic (Jeong et al., 2021). Moreover, the metaverse can assist people’s learning, serious games, and preschool teaching. Indeed, the metaverse can contribute to education by providing immersion, simulation of realistic scenes to better explain and clarify the courses, and effective teaching without harmful real-world experiences (Xu et al., 2021). Learning in a 3D virtual environment is more attractive to students compared to a traditional classroom. One developer reflected on the education evolution he had witnessed, indicating that “I learned geometry in 2D textbook modality, and I struggled. Fast forward to today and I watched my daughter learn *via* theatricals which had much more engaging visuals that changed the learning experience for her”^2^. “The immersive nature of these technologies and the potential for education, especially for students who are not necessarily engaged in school, is huge,” said a panel participant, Ariam Mogos, a tech lead and educator at Stanford D. School. “Where I think we have to be cautious is thinking about the role of educators and education administrators. The metaverse is composed of many different technologies and tools. To create an effective engaging learning experience, educators have to be versed in these technologies and understand how to design with them”^1^. The developer also predicted that “We’re going to have deeper immersion into virtual simulated worlds that we’re not just looking upon, but we are within them. The learning opportunities within that journey are endless”. He added that: “Studying anatomy, you are using it to look at the body for deeper understanding in ways that 2D could not do. That’s an amazing capability. We’re seeing those use cases today. And if you are an engineering or architectural student, those are use cases where it works. In other cases, we’ll have to be smart to apply it in true value add ways “^2^. Therefore, in metaverse learning, the virtual is expected to become real and work for all users.

Therefore, learners must know these 3D virtual environments and determine the possibility of improving applications and educational activities. Moreover, it is important to be able to design and provide these educational accomplishments within these three-dimensional backgrounds. As Mogos said, as the existence of novel tools becomes more ubiquitous, investment is required to provide learners of all stages with support and instruction so they can lead from a strong level of understanding.

Recent cutting-edge research is based on the development of a 3D VLE. Among these studies, Li (2022) proposed a platform for studying online in virtual learning, in which students could be in parallel with classroom courses in the pre-pandemic era. This platform improves the skills of students in digital learning. Literature reviews have also highlighted the immersion of diverse students from different domains in virtual reality platforms, including Evangelista (2021), who reported on medical students, and Canessa and Tenze (2021), who developed a new medium, OpenEyA, based on YouTube. This platform permits archiving and sharing traditional of courses for students of mathematics and physics without human interference or expensive management. Arif (2021) described the use of virtual reality in teaching in engineering, in which a connection assessment unit was planned for a cave automatic virtual environment-based system, called “Projection VR” and installed at the NED University Virtual Reality Center. Alexa et al. (2022) assessed the spread of changes, access, and integration of new technologies in online learning during the coronavirus-2019 pandemic in 134 engineering students at the Technical University of Lasi. In their study, 80.6% of students reported having resources to study in virtual classrooms and the ability to work in virtual platforms. Online learning and the success of these platforms in different levels of students were also reported by other studies such as those by Kalman et al. (2020) and Giray (2021). Pletz (2021) reported the good experience of 15 people from 13 organizations using immersive virtual reality platforms for education. The results reported by Nubi (2022) justified the importance of virtual learning in Africa as a mandatory pedagogical system during the pandemic.

The design of an intelligent system for meta-learning based on artificial intelligence using the metaverse was based on a 3D virtual environment, with animated characters based on powerful avatars. It contained personal electronic support, which provided the learners with sufficient flexibility that allowed them to learn without pressure, which required them to be present in specific places and specific times within a large group of learners. These advantages make our work novel and distinctive compared to related previous work.

## Description of the proposed system

In this study, we designed and developed an intelligent system for meta-learning based on artificial intelligence using the metaverse. This system is an attractive and self-configurable virtual environment, with animated characters based on powerful avatars.

### Architecture of the proposed intelligent system

**FIGURE 1:**
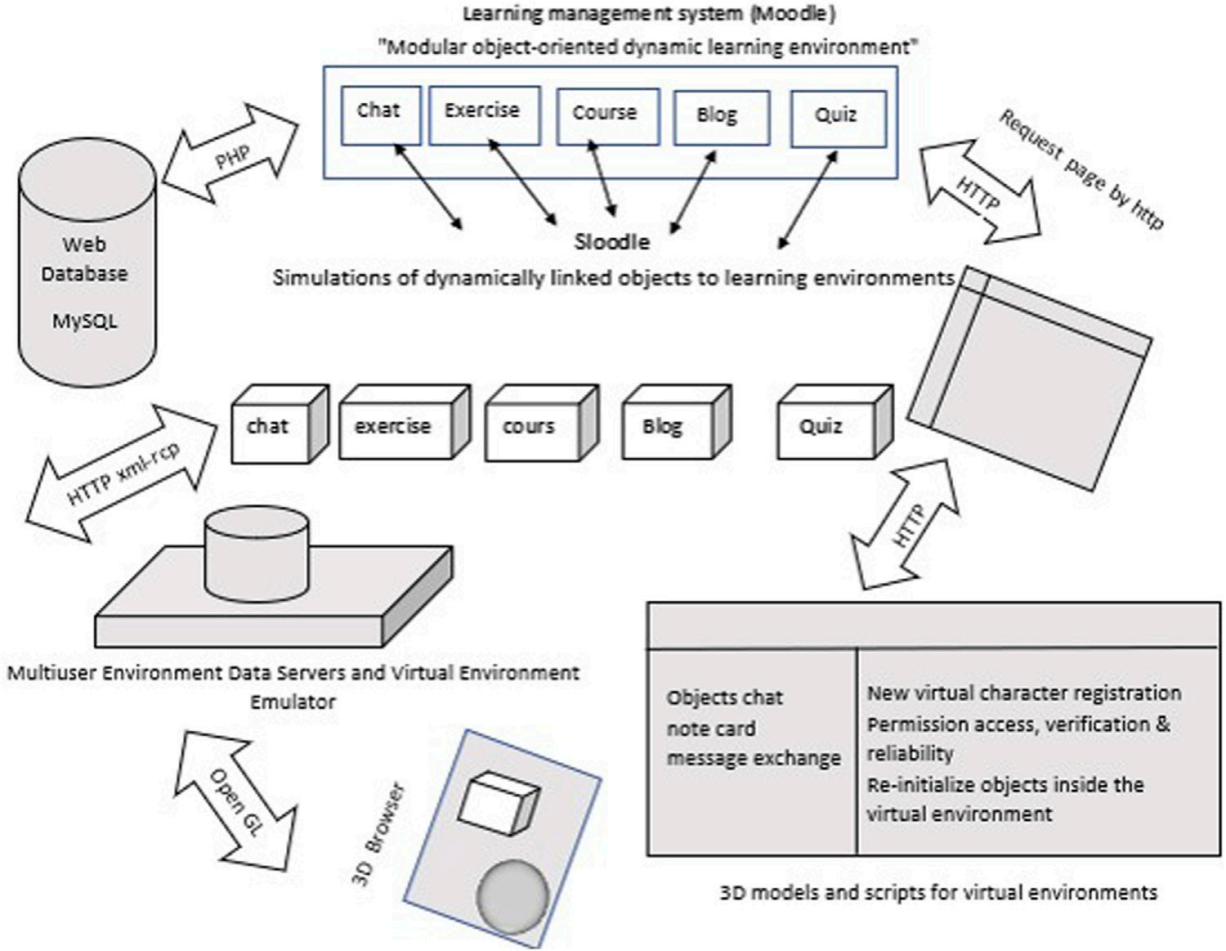
System block level diagram.

**FIGURE 2:**
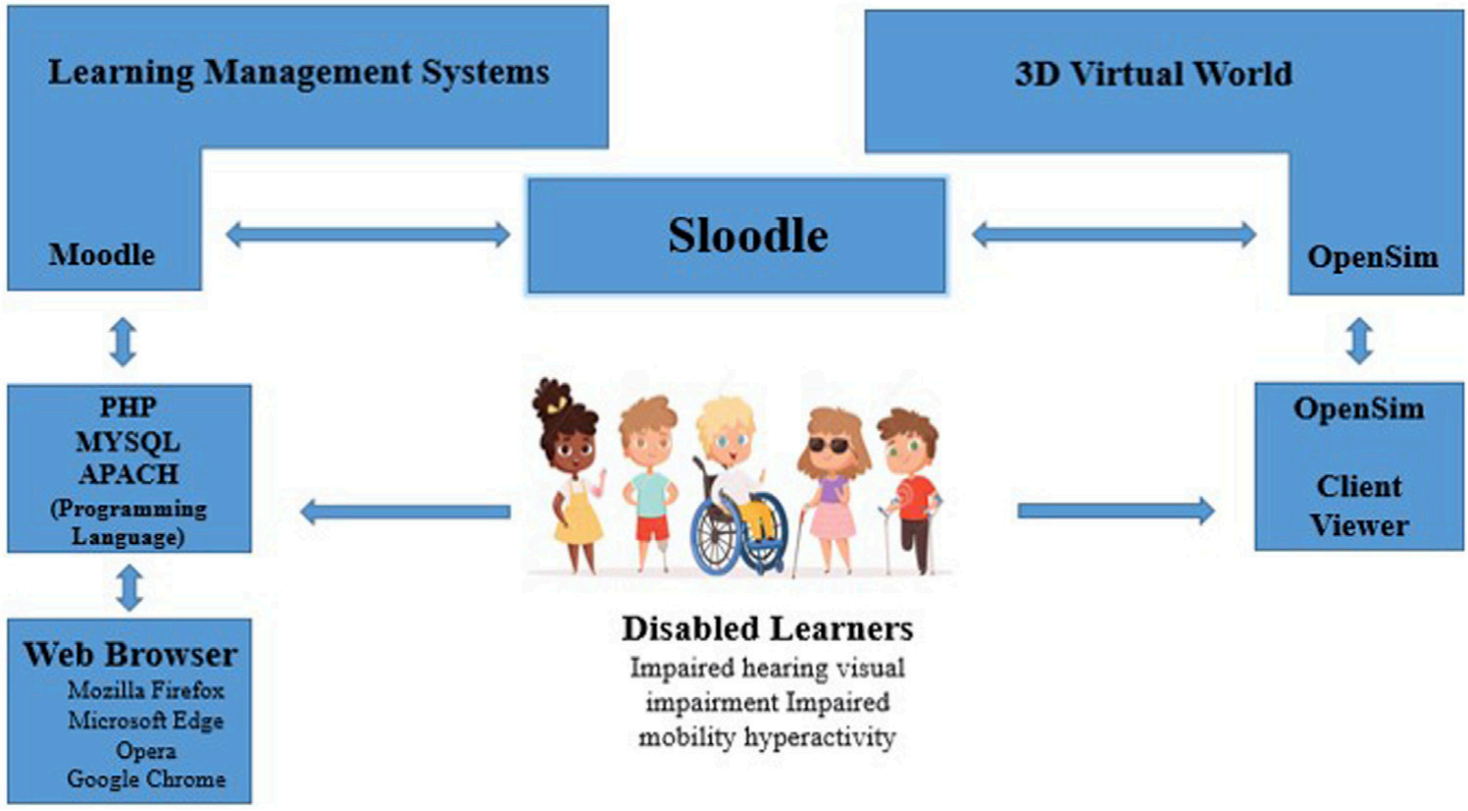
Architecture of the proposed intelligent system.

Meeting the requirements required a flexible design, as shown in Figure 1. The architecture of the intelligent system comprised three components. The first component was the Open Simulator, which included the world of virtual educational metaverse learning. This server allowed us to create, design, and emulate the metaverse. The second component was the Sloodle technology which was the bridge that connected the metaverse. The third component was Moodle, which represented the concept of e-learning. These components are open-source software that are widely used in educational institutions. As part of the proposed intelligent system, the names of students with disabilities, including motor disabilities, hearing impairments, hyperactivity, dyslexia, dyscalculia, etc., and their teachers were linked to the same user in Moodle through email services as virtual characters in the Open Sim emulator, which must be entered from the metaverse into the account. By using the Sloodle technology, students with disabilities and their teachers have access to Moodle materials and activities. Moreover, the architecture of the proposed system was practical, with access to the various technical tools and Sloodle management systems available in the metaverse. A system block level diagram is presented in detail in Figure 1; the different processing stages of the system with their implementation details and the diverse techniques used, as shown in Figure 2.

### Steps of system implementation

The metaverse in the system was built according to the archive files of the simulation program with special extensions (Oar, iar) that were downloaded from the folders of the simulation program. All the basic functions related to Sloodle were implemented, including registering students in Moodle inside the metaverse using the registration tool. Based on the analysis and design proposal of the system, the following steps were implemented:

#### Step 1: Configuration of the Moodle settings

The virtual class was first created in Moodle, as well as the activities of the learning management system: chat rooms, assignments, exercises, glossary, and others related to 3D objects inside the metaverse that is built and developed. This stage of implementation enabled teachers to transform and transfer their educational content to the virtual 3D classroom. For example, teachers could prepare a glossary of terms, create chat rooms for specific subjects, and define specific exercises and assignments to students inside these rooms.

#### Step 2: Constructing a virtual school building

A school was then built with different rooms or classes. For example, a room for presentations, another for assignments and exercises, and another for discussion in a specific course. Next to each room, information was available regarding the tool used inside the room.

#### Step 3: Configuring the Sloodle settings

Next, the files for the technology settings were included among the Moodle files for supervisors to add to the technology activities from inside Moodle. Both were then merged with the metaverse.

#### Step 4: Creating technical tools for Sloodle

Tools were then selected and their settings configured. For example, we choose the registration booth to support the registration of virtual characters. The presenter tool to support the presentations was set up in the Moodle chat room tool (Web-Intercom) to connect chat rooms in the two virtual environments and other tools of Sloodle.

#### Step 5: Completing the configuration of all Sloodle tools

Finally, all tools were linked to Moodle and were ready to perform their functions.

Although an innovative method, the limitations of the 3D VLE for students with disabilities were as follows:

1) Teachers and students with disabilities must be trained to use the system and periodically monitored.
2) Although the set-up cost is low because of the open-source software, it requires good specifications in terms of hardware and physical equipment, which is compatible with the nature of the system, such as the availability of computers with appropriate accuracy to display the 3D VLE.
3) When the system is configured in the network mode, students with disabilities require an Internet connection to access the virtual 3D classroom.
4) In the standalone mode, Moodle must be configured as a local host server so it can be easily linked to a 3D virtual environment system and benefit from Sloodle, which is available within.

### Sloodle functions

✔ Controller: The controller is created in Moodle; it allows the use of Sloodle in the curriculum and controls the licensing of objects. The controller also permits the supervisor of Moodle responsible for the curriculum to control the objects of the metaverse and determine who can access the courses related to Moodle.
✔ Web-Intercom: OpenSim connects to the chat room and all interactions that take place in the metaverse are recorded in Moodle.
✔ Registration: Students’ virtual characters are linked to user accounts in Moodle registering their virtual characters inside the metaverse.
✔ Quiz Chair: Through this tool, tasks, 3D models, and tests are performed. Grades can be determined by referring to the Moodle grade book.
✔ Choice Tool: Students can vote in the metaverse as well as in Moodle.
✔ Presenter: This function can display PDF files, web pages, and videos.
✔ MetaGlossary Tool: This tool enables users to identify the terms included in Moodle. Students or teachers only have to enter the word “/def” followed by the name of the term for which they want to search, which is registered in the glossary, to provide immediate results.

## Experimental results

**FIGURE 3:**
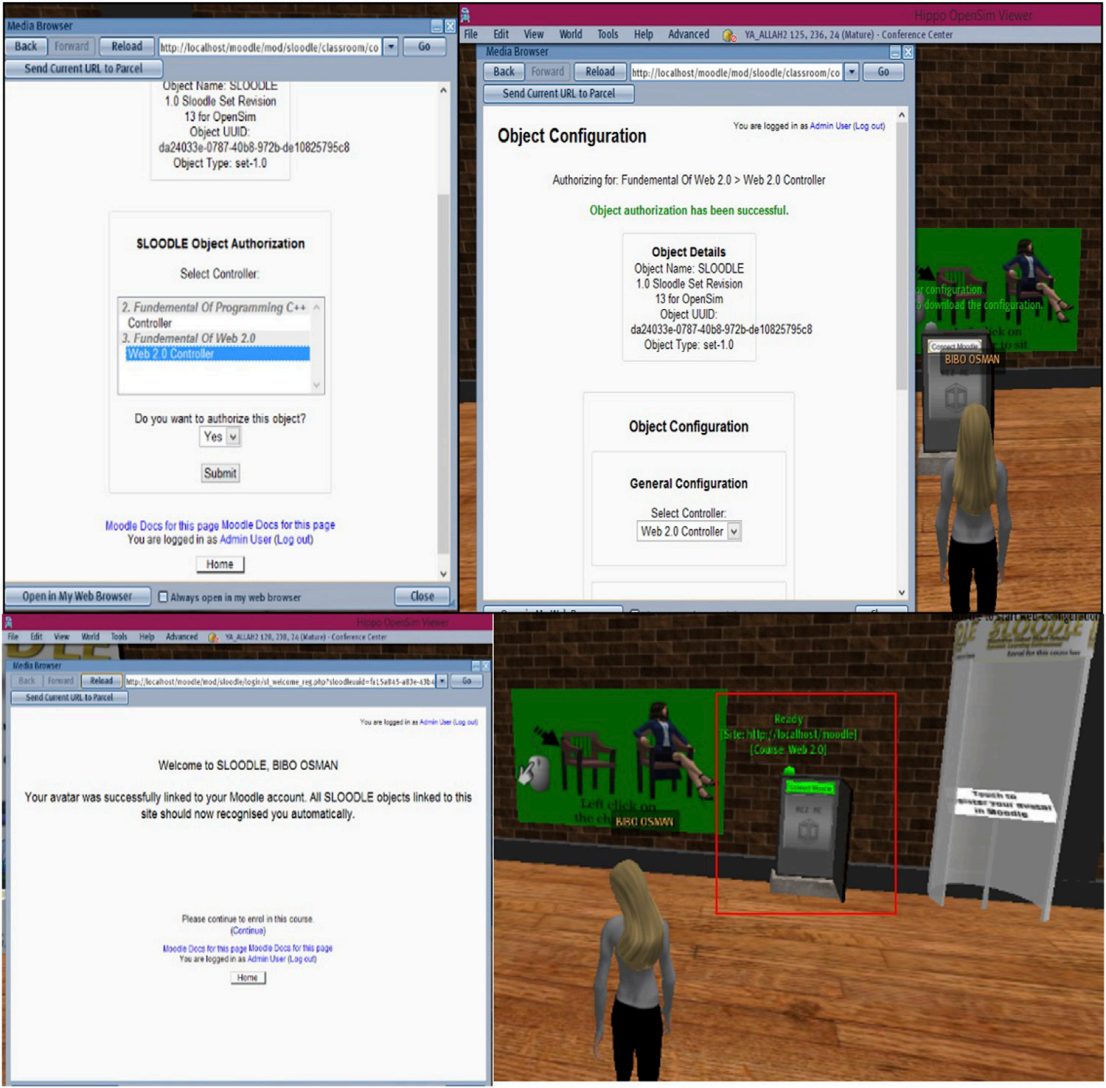
Inscription process for the intelligent system of virtual learning.

**FIGURE 4:**
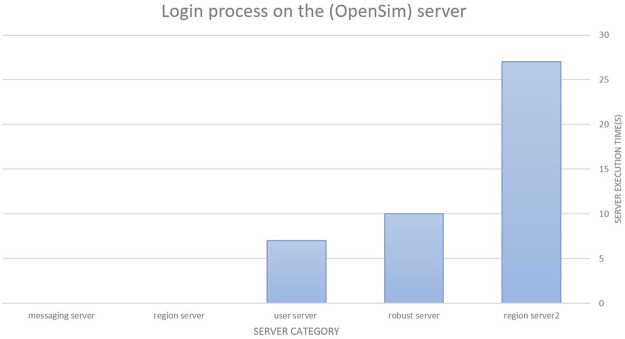
Login process on the Open Simulator server.

The first step of our study was the installation and configuration of an efficient platform to provide training and help for practical use. Users were required to log in to this platform to access the virtual courses (Figure 3). We evaluated the performance of the Open Simulator in both grid and standalone modes. With a single mode, it is easy to build the environment or virtual world to practice the metaverse for learning, whereas several designers have offered to reproduce their areas (regions) in Open Simulator. With these advantages, the single mode is considered a presentation tool for virtual environments. The standalone mode is easy to set up and configure. In contrast, the grid mode is an operating model that creates virtual environments like the world environment. The metaverse distributes the OpenSim Server regions, which comprise a large, virtualized environment that includes many regions and can be remotely navigated with features (teleporting). These modes were evaluated based on the login times. The results showed the environment times in the standalone and grid modes of 12 s and 16 s, respectively, demonstrating the robustness of the proposed platform. Figure 4 shows the login times. Open Simulator performed the following actions to verify the user/virtual: Access from the client to the server user (sending username and password) is needed according to the following steps:

1) Access from the user server to the region server where the client ID is sent to be connected.
2) Access from the user server to the robust server where the request is made regarding characteristics including the virtual appearance and information object files for the default environment (inventory).
3) Access from the user server to the messaging server, which informs the user that the login process is complete.

The steps take a few seconds; more specifically, the server takes 6 s to add a row for the region, about 19 s to update the appearance of the virtual character in the region server, about 8 s to prepare the object files with the virtual environment (inventory) on the robust server, and about 7 s to respond to the client’s authentication; i.e., login time (which can be reduced in the future).

The system was tested by 50 learners with disabilities. According to the results of the *t*-tests for independent samples, we measured the significance of the difference between the mean of two independent samples, as this test included two types of variables: the aggregation variable, which included two independent samples, and the test variable (a variable test), which included the study variable. We measured the effects of traditional tests and tests in the 3D virtual environment using the test chair tool to evaluate students with disabilities in primary school. In this step, the distribution of the test variable was assumed to be normal for each sample in the aggregation variable. This test was used in two cases:

✔ Assuming that the differences between the two samples were equal.
✔ Assuming that the variance of the two samples was unequal.

A test was conducted in the mathematics course after preparing the content of the course in Moodle and creating a course link with a 3D VLE using Sloodle. Next, we configured the course with the Sloodle package. The students with disabilities were divided into two equal groups. Twenty-five students with disabilities took the test using the traditional method, while 25 students with disabilities took the test using the chair test tool, which is one of the most important tools in the Sloodle technology. The following steps were conducted to perform the *t*-tests:

### Determining the level of significance (0.5)

The summed variance S2 was calculated as follows:

$$S_{p}=\sqrt{\frac{\left(n_{1}-1\right)S_{1}^{2}+\left(n_{2}-1\right)S_{2}^{2}}{n_{1}+n_{2}-2}}$$ (1)

$$S^{2}=\frac{\left(25-1\right)8^{2}+\left(25-1\right)6^{2}}{\left(25-1\right)+\left(25-1\right)}=\frac{2400}{48}=50$$

with

n1 = the sample size (i.e., the number of students) for the first sample
n2 = the sample size (i.e., the number of students) for the second sample
s1 = the standard deviation of the first sample
s2 = the standard deviation of the second sample

The *t*-value was calculated as follows in Eq. 2:

$$t=\frac{{\bar{X}}_{1}-{\bar{X}}_{2}}{\sqrt{\left(\frac{\left(N_{1}-1\right)S_{1}^{2}+\left(N_{2}-1\right)S_{2}^{2}}{N_{1}+N_{2}-2}\right)}\left(\frac{1}{N_{1}}+\frac{1}{N_{2}}\right)}$$ (2)

where

x1 = the average score of the students
x2 = the average score of the female students
n1 = the sample size (i.e., the number of students) for the first sample
n2 = sample size (i.e., the number of students) for the second sample
s1 = the standard deviation of the first sample
s2 = the standard deviation of the second sample
x = the compound standard deviation

To evaluate the proposed system, we performed *t*-tests for two independent samples to determine the significance of the differences between the averages of the experimental (students with disabilities using 3D VLE) and control (students with disabilities using traditional learning) groups in the cognitive post-measurement of the mathematics course, as shown in Table 1.

**TABLE 1:** Significance of the differences in the mean scores of the experimental and control groups.

Groups	SMA	Standard deviation	Sample (n)	Values (t)	Value (sig)	Indication level
Experimental	15.50	0.688	25	15.471	0.0001	Function
Control group	11.15	1.226	25			

We performed *t*-tests for two independent samples to determine the significance of the differences between the averages of the experimental and control groups in the post-measurement of the performance test for the skills of a mathematics course. The results are presented in Table 2.

**TABLE 2:** Significance of the differences in the averages of the experimental and control groups.

Groups	SMA	Standard deviation	Sample (n)	Value (t)	Value (sig)	Indication level
Experimental	101.30	2.203	25	21,006	0.0001	Function
Control group	74.40	6.012	25			

We performed *t*-tests for two independent samples to determine the significance of the differences between the averages of the experimental and control groups in the post-measurement of the skills of practical experiments, as shown in Table 3.

**TABLE 3:** Significance of the differences in the averages of the experimental and control groups.

Groups	SMA	Standard deviation	Sample (n)	Value (t)	Value (sig)	Indication level
Experimental	116.80	2.331	25	25.067	0.0001	Function
Control group	85.55	5.781	25			

Moreover, we performed *t*-tests of the linked samples to determine the significance of the differences between the mean scores of the experimental groups and the specific degree of mastery (80%) in the skill test.

We also performed *t*-tests of the linked samples to determine the significance of the differences between the mean scores of the control group and the specific degree of proficiency (80%) in the skill test for practical experience skills. The results are shown in Table 5.

**FIGURE 5:**
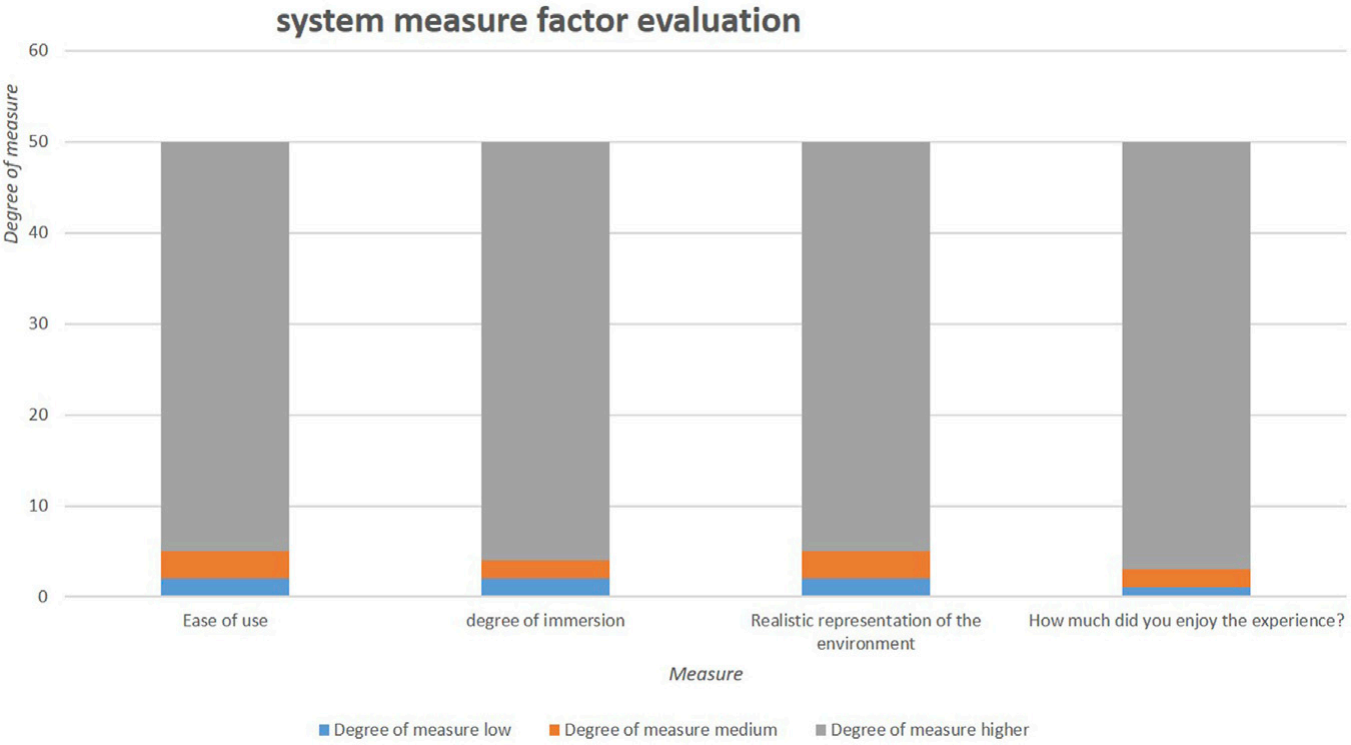
User experience evaluation.

The system was tested by 50 students with disabilities according to a set of factors to give a true evaluation, as illustrated in Figure 5.

## Discussion

**FIGURE 6:**
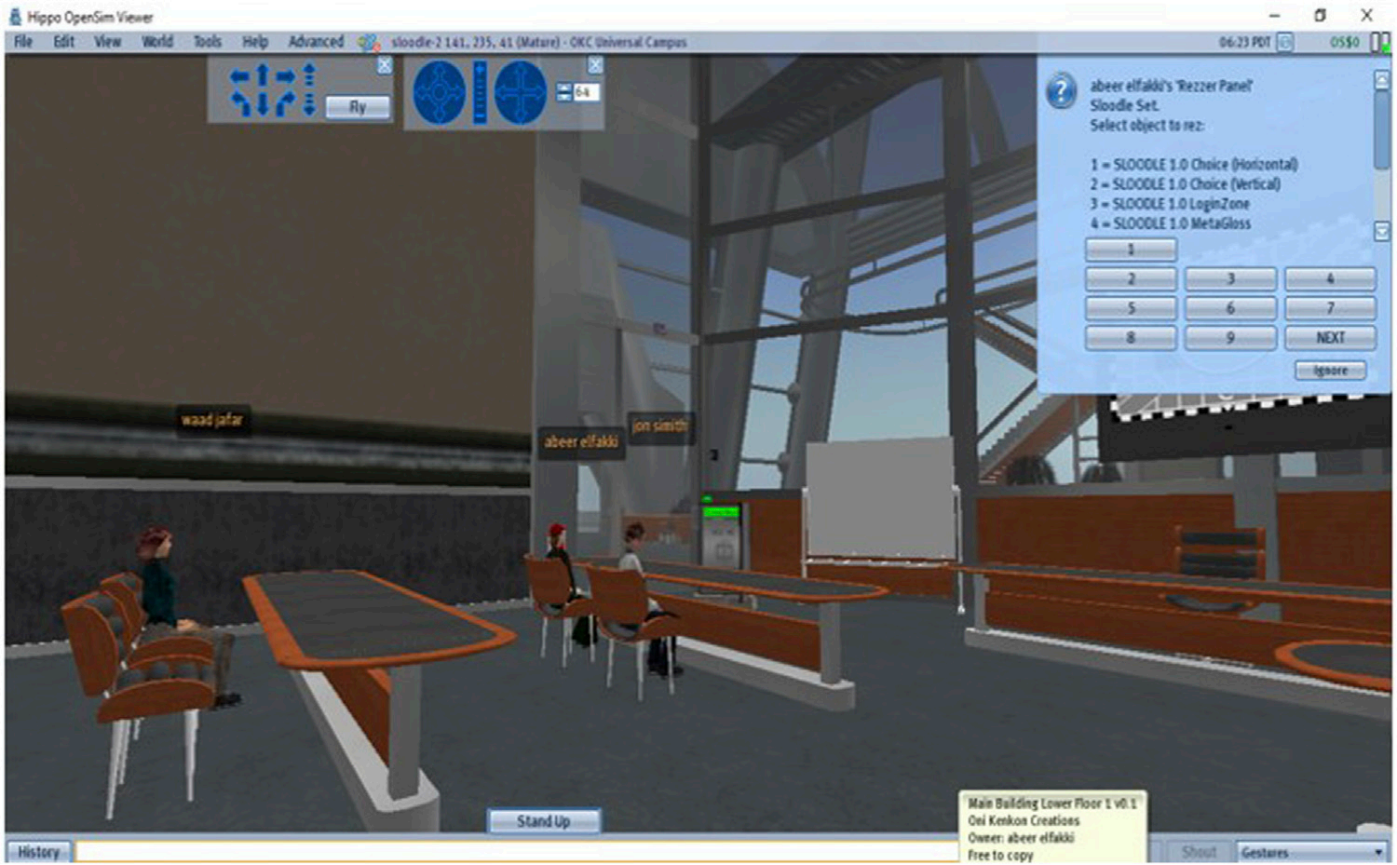
A 3D virtual learning environment.

**FIGURE 7:**
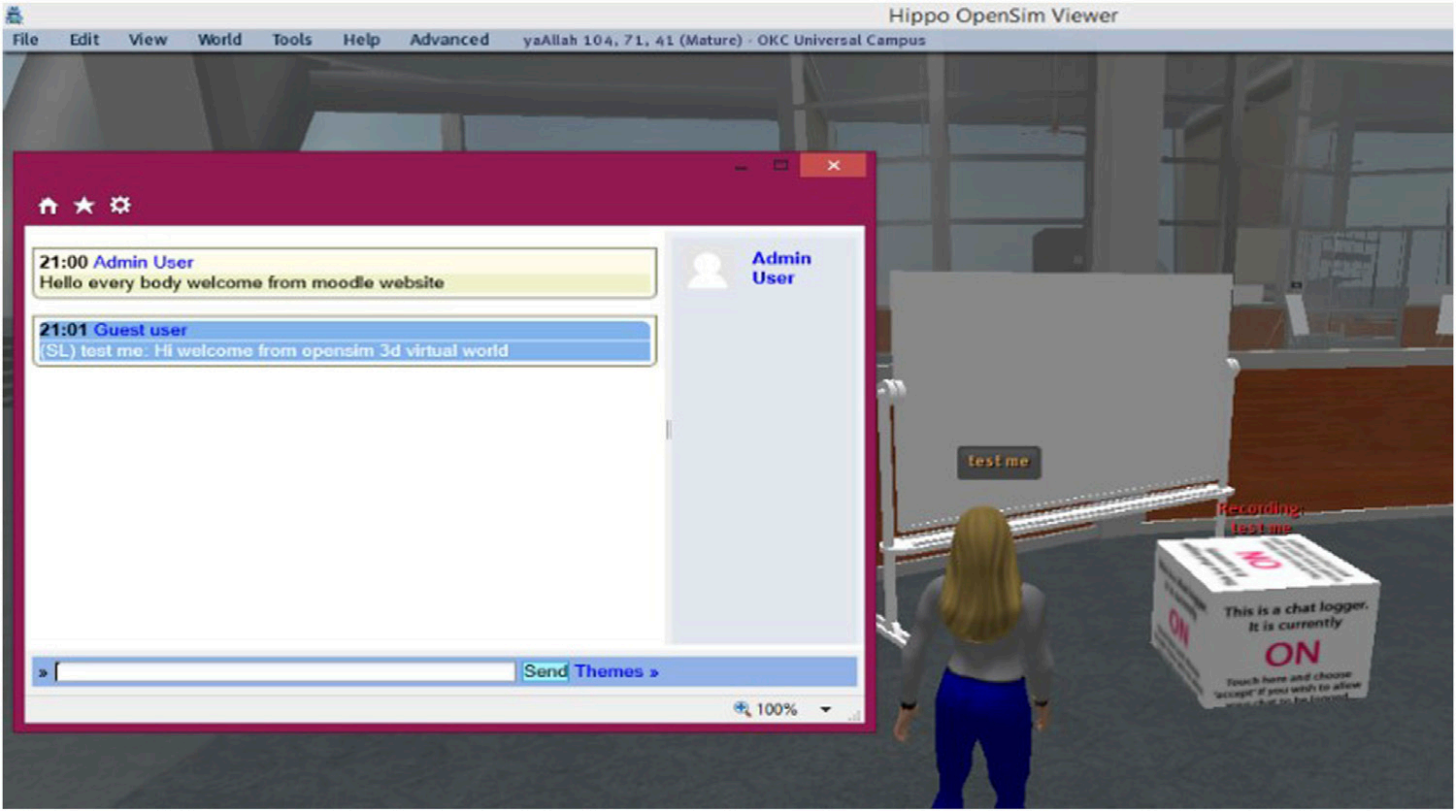
Interface for communication.

Our intelligent system provided not only personal electronic support, in a virtual learning 3D environment based on the metaverse as presented in Figure 6 but also provided the learners with sufficient flexibility to allow them to learn without pressure. This advantage permitted them to be present in specific places and times within a large group of learners, while social electronic support was based on the interaction between learners; thus, this tool can be used to shape and build their knowledge, which helps in building educational content. The heterogeneity of learners and the provision of a non-typical learning environment characterized by interactive, immediate, integrated, and cosmic students allow the users to represent the virtual community with their avatar and enable them to start their life as a resident of the virtual community. This method of learning considers strong visibility not only on topics of expertise but also on innovative learning devices compared to Zoom, Microsoft Teams, or Blackboard. Moreover, in the presented work, we developed a system for sharing progress (offline/online) between the users of the application and sharing messages (Figure 7). This system provides excellent collaboration tools for the 3D learning environment. With the proposed intelligent system, all users can communicate with other residents using voice chat and SMS messages. Furthermore, they can receive calls from outside the virtual community, as well as its residents. Students with disabilities can also travel in its various parts and socialize with other residents, participate in individual and group activities and conferences, and create virtual properties and services.

Table 1 presents the significance of the differences between the mean scores of the experimental and control groups in the post-application of the cognitive test of practical experience skills. Table 1 shows the high level of achievement of the experimental group of students using the 3D VLE compared to the control group using the traditional method. The average scores of the experimental and control groups were 15.50 and 11.50, respectively. The “t” value was 15,471, with a statistically significant p-value of 0.0001. Accordingly, the first hypothesis is accepted.

Table 2 shows the significance of the differences between the averages of the experimental and control groups in the post-measurement of the performance test for the skills of a mathematics course. Among the students with disabilities, those using the 3D VLE showed a high level of achievement compared to those using the traditional method. The value of “t” was 21,006, with a statistically significant p-value of 0.0001. Consequently, the second hypothesis is accepted.

Table 3 presents the significance of the differences between the averages of the experimental and control groups in the post-measurement of the skills of a mathematics course. Table 3 shows the high level of achievement of the experimental group of students with disabilities using the 3D VLE compared to the control group using the traditional learning method. The value of “t” was 25,067, with a statistically significant p-value of 0.0001 for the experimental group with the highest average. Thus, the third hypothesis is accepted.

Table 4 shows that the skill level of the experimental group students increases more than the specified degree of proficiency (80%), as the average scores of the experimental group in the dimensional measurement are 98% (>80%), and with a “t” value of 40,326. Moreover, the sig value is 0.0001, which is statistically significant at the level of α = 0.05. Hence, the fourth hypothesis is accepted.

**TABLE 4:** Significance of the differences between the average scores of the experimental groups and the specific degree of proficiency.

Groups	SMA	Standard deviation	Sample (n)	Value (t)	Value (sig)	Indication level
Pre-measuring process	116.80	2.331	25	40.326	0.0001	Function
Proficiency score (80)%	98.00	0.001	25			

Table 5 shows that the skill level of the students in the control group decreases and does not reach the required level. The average post-measurement score of the control group was 70%, which was <80%, and the value of “t” is 13,406. The sig value is 0.0001, which is statistically significant at the α = 0.05 level. As a result, the fifth hypothesis was rejected.

**TABLE 5:** Differences in the average scores of the control groups and a specific degree of proficiency of 80%.

Groups	SMA	Standard deviation (Z)	Sample (n)	Value (t)	Value (sig)	Indication level
Telemetry	85.55	5.781	25	13.406	0.0001	Function
Proficiency score (80)%	70.00	0.000	25			

The system is tested by 50 users and according to a set of factors. To give the true evaluation the results are shown in Table 6.

**TABLE 6:** Factors measured for system evaluation.

No	Measure	Degree of measure		
1	Ease of use	Ease	Medium	Hard
2	Degree of immersion	Bad	Good	Higher
3	Realistic representation of environment	Low	Medium	Higher
4	How much did you enjoy the experience?	Bad	Good	Higher

Figure 5 demonstrates that the proposed system for metaverse learning has excellent access and acceptance for all students in general, and especially for disabled people, in terms of ease of use and a high degree of immersion, particularly regarding the use of special materials (glasses, headphones, etc.), thus demonstrating the simplicity of using this environment not only for teaching and learning but also for evaluation (exams and quizzes).

**FIGURE 8:**
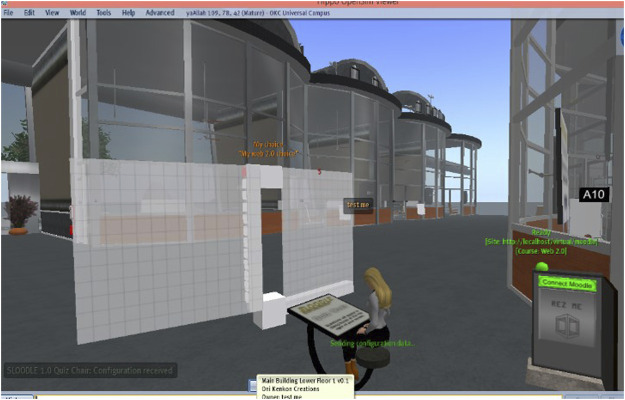
Interface for exams.

The proposed system makes the virtual teaching backgrounds important for everyday life with a low cost of studying and teaching. This system also encourages learners because it focuses on the most significant and immersive content to not only create an immersive learning experience but also provide the opportunity to learn and study in any location. It also encourages learners to transcend the state of passive reception of information and to move towards active participation in the education process. Even quizzes and exams can be completed virtually by students (Figure 8), especially those with disabilities that prevent them from easily moving from one place to another.

Table 7 compares the proposed approach to the state-of-the-art of related work in terms of age, number, and sex of disabled students practicing virtual learning. Moreover, we describe the different technologies used and the purpose of each approach.

**TABLE 7:** Comparisons between the proposed approach and related systems.

Ref.	Target participants	Age of participants	Personalized/General	Assistive technology used	Type of technology used	Purpose
Laabidi et al. (2014)	Primary school pupils with disabilities	—	General	Screen reading software/Screen magnification software/Braille display	Accessible version “Moodle Acc+	Online learning environment for persons with disabilities
Srivastava and Haider, (2020)	Elementary school dyslexic children	6–9 years	Personalized	Personalized e-learning model	Preparing knowledge base, Learning Models	Learning content for each dyslexic student based on their cognitive traits
Rello et al. (2014)	48 (29 girls, 19 boys) dyslexic children	—	General	Game for iPad	Onscreen text readability	Improving spelling and reading of dyslexic children
Mejía Correder et al. (2015)	Spanish speaking University students	—	General	Web based learning management multisensory system technique LS (interface with Moodle)	Multisensory technique	Providing integrated tool with Moodle for improved learning access of Spanish speaking dyslexic University students, assisting tool for teachers
Habib et al. (2012)	12 adults with dyslexia-in semi structured interviews, 24 adults (12 with dyslexia and 12 without dyslexia} in questionnaire survey	19–36 years	General	VLEs: fronter-eyetracking device, talking word processes	Qualitative data: semi structured interviews, quantitative data, questionnaire	Providing better writing, time saving (spellchecker and grammar checker highlight mistakes), identification and correction of errors
Kalyvioti and Mikropoulos, (2012)	Control group: 7 students without dyslexia (3 male, 4 female) Experimental group: 7 students with dyslexia (4 male,3 female)	Undergraduate students of University of Ioannina, Greece	General	VIRDA-MS (Virtual Reality Dyslexia Assessment-Memory Screening)	Virtual reality	Helping to manage daily memory challenges, tackling short-term memory and long-term memory
Proposed approach	50 primary school disabled students	6–9 years	General	VLEs with Sloodle technology having many tools: (presenter, quiz chair, choice tool, meta-glossary tool, web-intercom, registration- controller)	Virtual reality	To help disabled student in their daily class activities and learning process management
	Control group: 25 disabled students traditionally Taking the test Experimental group: 25 disabled students taking the test using quiz chair Sloodle					

## Conclusion

The proposed project was based mainly on the real-time teaching, learning, and training of people in virtual worlds and 3D spaces, especially in the current context of the COVID-19 pandemic. Disabled students can easily access more varied materials to help them interact with the 3D virtual world. Indeed, teachers and students will no longer need to be physically present in the classroom or even in the same country for learning or tests and exams. Thus, the metaverse may allow them to access these experiences as avatars. Meta-learning is characterized by flexibility and access to instant information sources from different parts of the world. The intelligent system in the present study provides educational platforms with the advantages of the learning management system Moodle, simulation based on Sloodle, and OpenSim software through the management of virtual environments. This system exchanges and transfers data between any two connected environments. The meta-learning system is very useful, rapid, effective, interesting, and acceptable, especially for people with disabilities. Depending on the scope of the proposed system requirements, the system must provide students with disabilities with a tool and technology/system that connects the emulator 3D VLE platform (OpenSim) to Moodle, which allows these students to access the 3D VLE through a standalone mode in a local network or by making the system available as an application on a large internal network or over the web. The system proposed in this study can easily create and manage Moodle educational content inside the 3D VLE and interact with different students with disabilities. The 3D VLE links the name of the user or virtual character in the Open Sim emulator to the same user in Moodle through the email service. Students with disabilities can practice several functions such as inviting friends to visit their virtual learning physical laboratory and 3D virtual environment; controlling the virtual personality and its appearance; and walking, flying, teleporting, sending group messages, and listening to group conversations. The 3D VLE provides a dedicated space for integrated audio conferencing and sound support. This intelligent system showed a significant difference (*α* = 0.05) between the mean scores of the experimental group of students with disabilities who used Sloodle and the control group of students with disabilities who used the traditional method in the post-measurement of the performance test for the mathematics course. The difference between the mean scores of the experimental group and the specific degree of proficiency (80%) was not statistically significant (p>0.05). However, the mean scores of the control group that used the traditional method and the specific degree of proficiency (80%) showed a statistically significant difference (p<0.05).

## Data Availability

The datasets presented in this article are not readily available due to concerns regarding participant/patient anonymity. Requests to access the datasets should be directed to the corresponding author.

## Footnotes

1
http://www.secondlife.com/

2
https://about.att.com/newsroom/2021/5G_metaverse.html
